# Prognostic role of podocalyxin-like protein expression in various cancers: A systematic review and meta-analysis

**DOI:** 10.18632/oncotarget.14199

**Published:** 2016-12-25

**Authors:** Jing Wang, Yongzhao Zhao, Ruizhao Qi, Xiaoning Zhu, Chenshen Huang, Sijin Cheng, Shengzhi Wang, Xiaolong Qi

**Affiliations:** ^1^ Department of Hepatobiliary Disease, Affiliated Traditional Chinese Medicine Hospital, Southwest Medical University, Luzhou, China; ^2^ School of Medicine, Tongji University, Shanghai, China; ^3^ Department of General Surgery, 302 Hospital of PLA, Beijing, China

**Keywords:** prognostic, podocalyxin-like protein, cancer, overall survival

## Abstract

Several studies were conducted to explore the prognostic significance of podocalyxin-like protein (PODXL) expression in various cancers, with contradictory. This study aims to summarize the prognostic significance of PODXL expression in cancers. PubMed, the Cochrane Library and Embase were completely retrieved. The prospective or retrospective studies focusing on the prognostic role of PODXL expression in cancers were eligible. The endpoints were overall survival (OS), disease-specific survival (DSS) and disease-free survival (DFS).12 studies involving a total of 5,309 patients were identified. The results indicated that high PODXL expression was significantly associated with worse OS when compared to the low PODXL expression (HR=1.76, 95%CI=1.53-2.04, p<0.00001; I^2^=41%, *p*=0.08). And similar results were detected in the subgroup analysis of analysis model, ethnicity, sample size, tumor type and antibody type. And the results also showed that high PODXL expression was obviously related to shorter DSS (HR=2.47, 95%CI=1.53-3.99, *p*=0.0002; I^2^=66%, *p*=0.03) and DFS (HR=2.12, 95%CI=1.58-2.85, p<0.00001; I^2^=19%, *p*=0.29). In conclusion, it was revealed that high PODXL expression is an unfavorable predictor of OS, DSS and DFS in patients with cancers, and high PODXL expression is a promising prognostic biomarker for cancers, especially for patients in European.

## INTRODUCTION

Podocalyxin-like protein (PODXL), an important transmembrane glycoprotein belonging to the CD34 family [1], is usually expressed on the apical surface of podocytes and glomerular epithelial cells [2]. However, it also expressed by breast epithelium [3], vascular [4] and haematopoietic progenitors [5]. The main function of PODXL was regulating the cell morphology and adhesion by the connections between the intracellular proteins and extracellular ligands [6–8].

Recently, several studies indicated that PODXL might be a predictor of prognosis in various cancers, such as colorectal cancer (CRC) [9–12], pancreatic cancer (PC) [13–15], glioma [16], esophageal and gastric adenocarcinoma (EGAC) [17, 18], urothelial bladder cancer (UBC) [19], breast cancer (BC) [20] and so on. The role of PODXL in tumorigenesis remains unclear, but it has been demonstrated to promote the tumor growth, invasion and metastasis [21, 22].

Although it was reported that PODXL expression might be associated with prognostic outcomes in patients with cancers, the dispute does exist. The study conducted by the *Larsson et al 2016* showed that high PODXL expression predicted worse overall survival (OS) in CRC when compared with the low PODXL expression in cohort II, however, no significant correlation between the PODXL expression and OS was observed in cohort III [11]. *Chijiiwa et al* covered that high PODXL expression was obviously related to the shorter disease free survival (DFS) in PC [14], but *Forse et al* reported that high PODXL expression was a favorable factor of prognosis in BC [20]. Besides the study conducted by *Heby et al* presented that high PODXL expression was evidently associated with worse recurrence-free survival (RFS) in cohort I. Nevertheless, no significant relationship was detected in patients in cohort II [13]. In view of the controversy, the systematic review and meta-analysis was performed to explore the prognostic role of PODXL expression in various cancers.

## RESULTS

### Literature search

**Figure 1:**
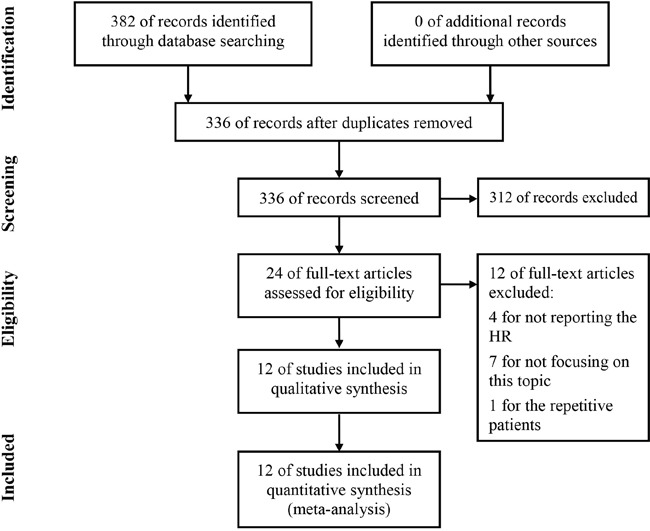
Flow diagram of study selection process

As shown in Figure 1, a total of 382 papers were identified and 46 duplicative papers were excluded. As for the remaining 336 papers, 312 were excluded by scanning the titles or abstracts. For the 24 potentially associated studies remained, the full-texts were carefully read. Among these 24 studies, 4 were excluded for insufficient datum to assess the HR of prognosis outcomes, and 7 were excluded for not focusing on the topic, and 1 were excluded because the included patients were covered in the other one study. At last, 12 studies involved 5,309 patients were eligible for this meta-analysis [9–20].

### Characteristics of included studies

As listed in Table 1, the twelve included studies contained sixteen cohorts involving 5,309 patients [9–20]. The percentage of males in the included studies varied from 46.0% to 77.2%. And the sample size was also different, varying from 70 patients to 775 patients. In term of ethnicity, nine studies focused on the European [9–13, 15, 17–19], one study focused on the Asian [14] and two studies focused on the North American [16, 20]. As for survival analysis, nine studies reported the OS [9–11, 13–16, 18, 19], four studies covered the DSS or CSS [9, 12, 17, 19], two studies reported the TTR [10, 18], three studies reported the DFS [10, 14, 20], one study covered the PFS [19], and one study reported the RFS [13]. In addition, four studies focused on the prognostic role of PODXL in CRC [9–12], one study focused on the glioma [16], one focused on the UBC [19], one paid attention to the BC [20], three focused on the PC [13–15], and two studies focused on the prognostic role of PODXL in EGAC [17, 18]. It must be said that three studies conducted by *Larsson et al* respectively focused on the same cohorts [9–11]. Besides, the HRs of prognosis were assessed with multivariate analysis in ten studies [9–13, 15, 17–20], while assessed with univariate analysis in the other two studies [14, 16]. Moreover, six studies used the polyclonal antibody to stain the PODXL [9–11, 13, 18, 19], one studies used the monoclonal antibody [14] and three studies used both polyclonal antibody and monoclonal antibody [12, 15, 17] but two studies did not report the antibody type [16, 20].

**Table 1:** Characteristics of included studies

Study	Year	Country	Ethnicity	Patients (n)	Male (%)	Outcome	Tumor	Analysis	Antibody
Larsson et al 2011 [9]	2011	Sweden	European	536	47.9	OS, CSS	CRC	M	P
Larsson et al 2012 [10]	2012	Sweden	European	576	50.3	OS, TTR, DFS	CRC	M	P
Binder et al 2013 [16]	2013	USA	North American	342	NR	OS	Glioma	U	NR
Boman et al 2013 (1) [19]	2013	Sweden	European	100	74.0	OS†	UBC	M	P
Boman et al 2013 (2) [19]	2013	Sweden	European	343	75.8	OS†, DSS, PFS∫	UBC	M	P
Forse et al 2013 [20]	2013	Canada	North American	698	0.0	DFS	BC	M	NR
Kaprio et al 2014 [12]	2014	Finland	European	775	45.0	DSS	CRC	M	P, Mo
Heby et al 2015 (1) [13]	2015	Sweden	European	63	46.0	RFS†, OS†	PAC	M	P
Heby et al 2015 (2) [13]	2015	Sweden	European	107	53.2	RFS†, OS†	PPC	M	P
Laitinen et al 2015 [17]	2015	Finland	European	266	51.5	DSS	GC	M	P, Mo
Borg et al 2016 [18]	2016	Sweden	European	171	77.2	TTR, OS	EGAC	M	P
Chijiiwa et al 2016 [14]	2016	Japan	Asian	70	NR	DFS, OS	PC	U	Mo
Larsson et al 2016 (1) [11]	2016	Sweden	European	533	47.3	OS	CRC	M	P
Larsson et al 2016 (2) [11]	2016	Sweden	European	259	49.0	OS	CRC	M	P
Larsson et al 2016 (3) [11]	2016	Sweden	European	310	49.0	OS	CRC	M	P
Saukkonen et al 2015 [15]	2015	Finland	European	168	NR	OS	PDAC	M	P, Mo

OS, overall survival; CSS, cancer-specific survival; TTR, time to recurrence; DFS, disease free survival; DSS, disease-specific survival; PFS, progression-free survival; RFS, recurrence-free survival; †, 5 years; ∫, 2 years; M, multivariate analysis; U, univariate analysis. CRC, colorectal cancer; UBC, urothelial bladder cancer; PC, pancreatic cancer; EGAC; BC, breast cancer. NR, not reporting. P, polyclonal antibody; Mo, monoclonal antibody.

### Meta-analysis of OS

**Figure 2:**
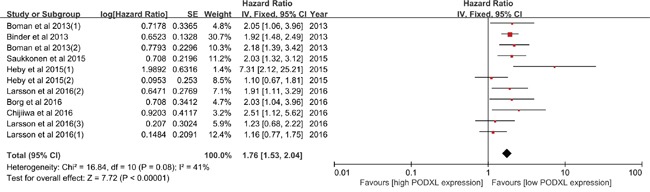
Meta-analysis of overall survival

Seven studies containing eleven cohorts were included into the pooled analysis. As shown in Figure 2, no obvious heterogeneity was detected (I^2^=41%, *p*=0.08), and fixed-effect model was used. The results presented that PODXL expression was distinctly associated with the OS (HR=1.76, 95%CI=1.53-2.04, *p*<0.00001), which indicated that high PODXL expression predicted worse OS compared to the low PODXL expression. In additions, the influence analysis was carried out and no decisive effect of included studies was observed (Supplementary Figure S1), and no significant bias among all included studies was detected by funnel plot (Supplementary Figure S2).

To explore the source of heterogeneity, the subgroup analysis of OS was conducted. As listed in Table 2, regarding the analysis model of included studies, it was shown that high PODXL expression was significantly correlated with the shorter OS when the included studies were assessed with multivariate analysis model (HR=1.67, 95%CI=1.40-1.99, *p*<0.00001; I^2^=48%, *p*=0.05), using fixed-effect model. And similar results were detected when the included studies were assessed with univariate analysis (HR=1.97, 95%CI=1.54-2.52, *p*<0.00001), with no heterogeneity (I^2^=0%, *p*=0.54). In terms of ethnicity, the results demonstrated that high PODXL expression was an unfavorable prognostic factor in cancers in European (HR=1.67, 95%CI=1.40-1.99, *p*<0.00001; I^2^=48%, *p*=0.05), in North American (HR=1.92, 95%CI=1.48-2.49, *p*<0.00001) and in Asian (HR=1.51, 95%CI=1.12-5.62, *p*=0.03). As for the subgroup analysis of sample size, PODXL expression was obviously related to the OS in patients with cancers both when sample size <300(HR=1.88, 95%CI=1.50-2.36, *p*<0.00001;I^2^=39%, *p*=0.13) and≥300(HR=1.62, 95%CI=1.20-2.18, *p*=0.002; I^2^=54%, *p*=0.09). With respect to tumor type, evident correlation between the PODXL expression and OS was observed. High PODXL expression might increase 35% risk of death compared with the low PODXL expression in CRC (HR=1.35, 95%CI=1.01-1.80, *p*=0.04; I^2^=9%, *p*=0.33), using fixed-effect model. And it was also shown that high PODXL expression was an adverse predictor of OS in patients with UBC (HR=2.14, 95%CI=1.47-3.10, *p*<0.0001; I^2^=0%, *p*=0.88). Similar results were observed in PC (HR=2.12, 95%CI=1.18-3.83, *p*=0.01; I^2^=69%, *p*=0.02) and EGAC (HR=2.03, 95%CI=1.04-3.96, *p*=0.04). Moreover, the results presented that high PODXL expression was distinctly associated with worse OS in patients with glioma (HR=1.92, 95%CI=1.48-2.49, *p*<0.00001). In the subgroup analysis of polyclonal antibody, the high PODXL expression was evidently related to shorter OS in cancers, using fixed-effect model (HR=1.67, 95%CI=1.40-1.99, *p*<0.00001; I^2^=48%, *p*=0.05), and similar result was detected in the subgroup analysis of monoclonal antibody (HR=2.13, 95%CI=1.46-3.11, *p*<0.0001; I^2^=0%, *p*=0.65).

**Table 2:** Subgroup analysis of overall survival

Survival analysis	Included cohorts	HR 95% CI	*p*	I^2^	*p* value for heterogeneity
**Analysis model**					
Multivariate	9	1.67 [1.40, 1.99]	<0.00001‡	48%	0.05
Univariate	2	1.97 [1.54, 2.52]	<0.00001‡	0%	0.54
**Ethnicity**					
European	9	1.67 [1.40, 1.99]	<0.00001‡	48%	0.05
North American	1	1.92 [1.48, 2.49]	<0.00001‡	NA	NA
Asian	1	1.51 [1.12, 5.62]	0.03‡	NA	NA
**Sample Size**					
<300	7	1.88 [1.50, 2.36]	<0.00001‡	39%	0.13
≥300	4	1.62 [1.20, 2.18]	0.002‡	54%	0.09
**Tumor**					
CRC	3	1.35 [1.01, 1.80]	0.04‡	9%	0.33
UBC	2	2.14 [1.47, 3.10]	<0.0001‡	0%	0.88
PC	4	2.12 [1.18, 3.83]	0.01‡	69%	0.02
EGAC	1	2.03 [1.04, 3.96]	0.04‡	NA	NA
Glioma	1	1.92 [1.48, 2.49]	<0.00001‡	NA	NA
**Antibody**					
Polyclonal antibody	9	1.67 [1.40, 1.99]	<0.00001‡	48%	0.005
Monoclonal antibody	2	2.13 [1.46, 3.11]	<0.0001‡	0%	0.65

OS, overall survival; NA, not applicable; CRC, colorectal cancer; UBC, urothelial bladder cancer; PC, pancreatic cancer; EGAC, esophageal and gastric adenocarcinoma; *p*<0.05, the difference was significant.

### Meta-analysis of DSS

**Figure 3:**
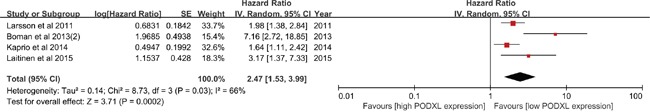
Meta-analysis of disease-specific survival

Four studies reported the DSS or CSS and were enrolled into the meta-analysis of DSS. As shown in Figure 3, in view of the obvious heterogeneity (I^2^=66%, *p*=0.03), the random-effect model was applied. And the results indicated that high PODXL expression was evidently correlated with the shorter DSS in cancers (HR=2.47, 95%CI=1.53-3.99, *p*=0.0002). Besides, no decisive effect was observed according to the influence analysis (Supplementary Figure S3), and no significant bias among all included studies was detected by funnel plot (Supplementary Figure S4).

### Meta-analysis of DFS

**Figure 4:**
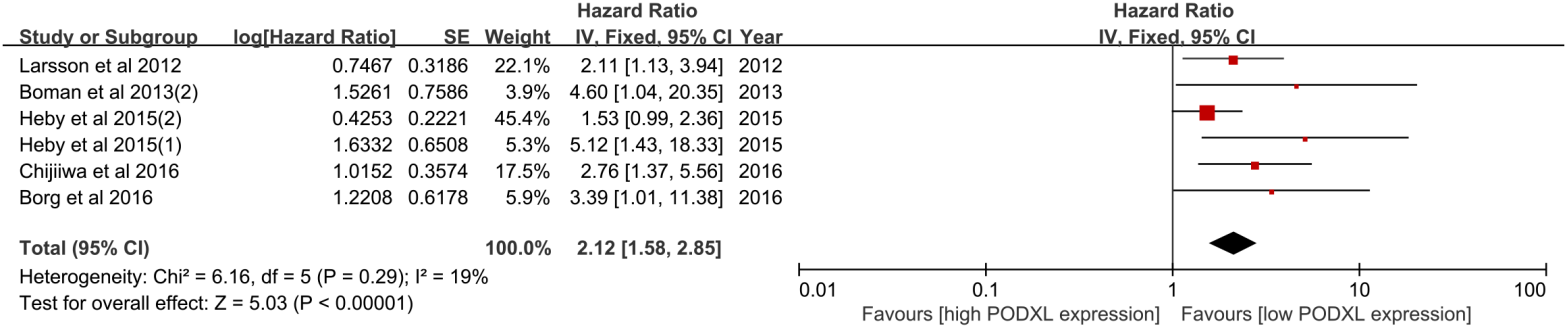
Meta-analysis of disease free survival

Two studies reported the TTR, three studies reported the DFS, one study reported the PFS and two studies reported the RFS. However, the study conducted by *Forse et al* was excluded for the apparent increase of heterogeneity (I^2^= 19% and I^2^=76%, respectively). The study conducted ty the *Larsson et al 2012* both covered the TTR and DFS, and the DFS was extracted and enrolled into the meta-analysis. Therefore, five studies including six cohorts were included into the meta-analysis of DFS. As shown in Figure 4, there was no obvious heterogeneity among the included studies (I^2^=19%, *p*=0.29). It was shown that high PODXL expression predicted shorter DFS when compared with the low PODXL expression in various cancers (HR=2.12, 95%CI=1.58-2.85, *p*<0.00001). In additions, no decisive effect was observed according to the influence analysis (Supplementary Figure S5), and no publication bias among all the included studies was detected by funnel plot (Supplementary Figure S6).

## DISCUSSION

Increasing evidences showed that PODXL expression was associated with prognosis in various cancers [9–20]. Furthermore, plenty of studies demonstrated that PODXL played a key role in the formation of primary tumors growth and metastasis [21, 22]. And *Lin et al* covered that PODXL might promote tumor formation and metastasis by activating the Rac1/Cdc42/cortactin signaling [23]. *Thomas et al* reported that PODXL is an E-/L-selectin ligand on colon carcinoma cells [7]. Therefore, more and more researchers focused on the role of PODXL in cancers. Nevertheless, the prognostic role of PODXL expression in cancers was controversial.

In our study, the results showed that high PODXL expression was significantly associated with worse OS compared with the low PODXL expression. And similar results were detected in the subgroup analysis of analysis model, ethnicity, sample size, tumor type and antibody type. The high PODXL expression was still obviously correlated with shorter OS when all the included studies were assessed with the multivariate analysis, which made the conclusion more convincing. It was also indicated that the correlation between the PODXL expression and OS remained distinct in European. Although similar results were observed both in Asian and North American, the results were short of reliability for the limited included studies. Therefore, to further explore the relationship between the PODXL expression and prognosis of cancers in Asian and North American, more relevant studies should be put into effect in Asian and North American. As for the sample size, the relationship between the PODXL expression and OS was distinct both when sample size≥300 and <300, which further confirmed the prognostic role of PODXL in various cancers. The results also showed that high PODXL expression predicted shorter OS in multiple tumors, especially in PC and CRC. In additions, although the *Larsson et al 2016* covered that no obvious correlation between the high PODXL expression and OS was observed in cohort I [11], which was inconsistent with the previous study [9], it was remained convincing for more patients assessed with multivariate analysis [11]. Besides, the antibody type influenced the prognostic role of PODXL expression in various cancers. In the study conducted by *Kaprio et al*, it was declared that high PODXL expression was an unfavorable predictor in CRC, and the patients with high PODXL expression predicted worst OS when the PODXL was stained by combined polyclonal antibody and monoclonal antibody [12]. Similarly, in the *Laitinen et al* study, the association between the PODXL expression and OS remained significant in multivariable analysis only when the polyclonal antibody was used [17]. Therefore, more prospective studies should be carried out to explore the antibody use in the immunohistochemical of PODXL.

As for the DSS, our study indicated that high PODXL expression predicted worse DSS when compared to the low PODXL expression. Combining the OS, the high PODXL expression became a promising predictor of prognosis in cancers and should be paid more attention to. In additions, it was revealed that PODXL expression was obviously associated with DFS, and high PODXL expression predicted worse DFS compared with the low PODXL expression. However, it should be noted that all the included studies in the meta-analysis of DSS was conducted in European, and main included studies in the pooled analysis of DFS also focused on the European. Therefore, the conclusion should be used cautiously in Asian. And more studies should be payed attention to the prognostic role of PODXL expression in cancers in Asian.

The highlighted strength of our study as follows: First, to the best knowledge of us, the study was the first systematic review and meta-analysis to explore the prognostic role of PODXL expression in various cancers, and the results contributed to the development of the research concerning the prognostic role of PODXL in cancers. Second, twelve studies with a relatively large population were finally included, hence, the results were convincing. Third, the comprehensive subgroup analyses were carried out, such as ethnicity, antibody type, tumor type and so on. Fourth, no obvious heterogeneity was observed in the meta-analysis of OS and DFS, therefore, the pooled effects were accurate.

Furthermore, several limitations of our study should be carefully considered. First, the conclusion of some tumors should be used with caution for the limited included studies, such as BC, glioma and so on. Second, the data was obtained from the published articles, so the individual data was unavailable. Third, the heterogeneity remained significant in the meta-analysis of DSS. And the accuracy of the results might be reduced for the use of random-effect model. Fourth, moderate publication bias might be existed for the reason that the researchers tended to report the positive results.

In conclusion, it was revealed that high PODXL expression is an unfavorable predictor of OS, DSS and DFS in various cancers, and high PODXL expression is a promising prognostic biomarker for cancers, especially for patients in European.

## MATERIALS AND METHODS

### Literature search strategy

PubMed, the Cochrane Library and Embase were comprehensively searched up to October 10, 2016. The search strategy was “((((Tumor) OR Neoplasm) OR Cancer) OR carcinoma) AND (((((podocalyxin-like 1) OR Podocalyxin-like protein) OR PODXL) OR Podocalyxin) OR Podocalyxin-like)”. All the retrieved papers were checked. And the reference lists were also checked. The obviously irrelevant articles were excluded by scanning the titles or abstracts. The remaining papers were then reviewed comprehensively by carefully reading the full text.

### Inclusion criteria

The studies meeting all the criteria should be included: 1) prospective or retrospective studies; 2) paying attention on the role of PODXL expression on the prognosis in various cancers; 3) enough data to get the hazard ratio (HR) for prognostic outcomes, along with their 95% confidence intervals (CIs) or *p* values; 4) studies published in English.

### Exclusion criteria

The exclusion criteria were as follows: 1) neither the prospective nor retrospective studies; 2) studies without enough data to get the HR; 3) studies not focusing on the role of PODXL expression on the prognosis in various cancers; 4) not published in English.

### Data extraction

All the manuscripts were independently reviewed by two investigators (Zhao Y and Wang J). The following data were carefully abstracted: first name of the first author, year of publication, country of the study, ethnicity, the numbers of patients, percentage of males, tumor type, survival outcomes, analysis model and antibody type. The HRs of prognostic outcomes obtained directly or indirectly from published articles were integrated in the meta-analysis according to the study conducted by *Tierney et al* [24]. If the multivariate analysis and univariate analysis were both applied in the study, the HR assessed with multivariate analysis was abstracted. If the PODXL was stained both by polyclonal antibody and monoclonal antibody, the datum of the former were extracted. Any disputes were discussed with the third investigator (Qi X).

### Statistical analysis

Meta-analysis was carried out by Review Manager Version 5.3 software. The prognosis outcomes were assessed using the HR, along with the corresponding 95% CI or *p* values. The prognosis outcomes mainly contained the OS, disease-specific survival (DSS), DFS, cancer-specific survival (CSS), progression-free survival (PFS), time to recurrence (TTR) or RFS. The heterogeneity was assessed by Cochran's Q test and Higgins I^2^ among included studies. And the heterogeneity should be considered when *p*< 0.05 and/or I^2^ > 50%, and the random-effect model was applied; if not, the fixed-effect model was used. Besides, the funnel plot was conducted to evaluate publication bias by Review Manager Version 5.3 software. The sensitivity analysis was conducted by Stata 12.0 to access the robustness of the results*. p* < 0.05 meant the correction was significant.

## SUPPLEMENTARY FIGURES AND TABLE




