# Supplementary material for: Prognostic role of podocalyxin-like protein expression in various cancers: A systematic review and meta-analysis
Source: Oncotarget. 2016 Dec 25;8(32):52457–64. doi: 10.18632/oncotarget.14199 (PMC5581042; doi:10.18632/oncotarget.14199)
Supplement: Supplementary file 1 [file oncotarget-08-52457-s001.pdf]

Prognostic role of podocalyxin-like protein expression in various cancers: A systematic review and meta-analysis

SUPPLEMENTARY FIGURES AND TABLE

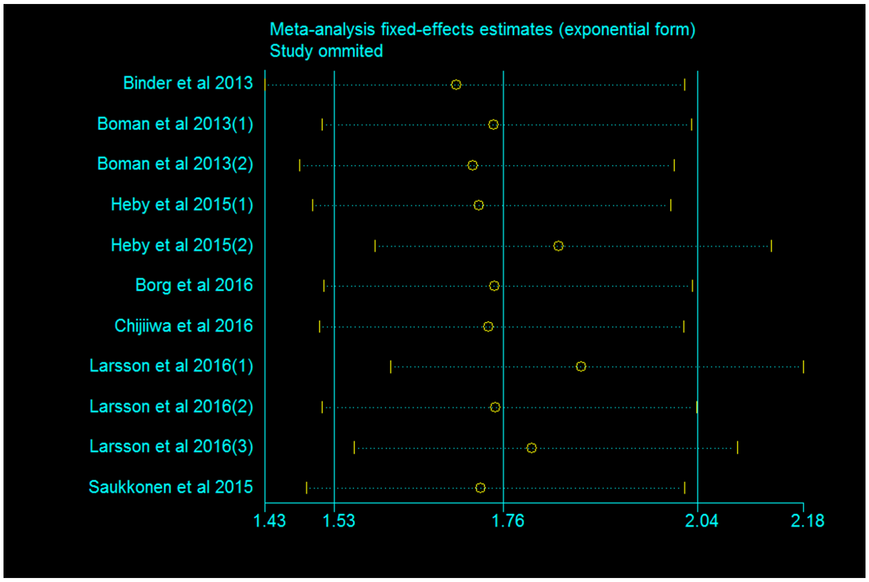

Supplementary Figure S1: Influence analysis of overall survival.

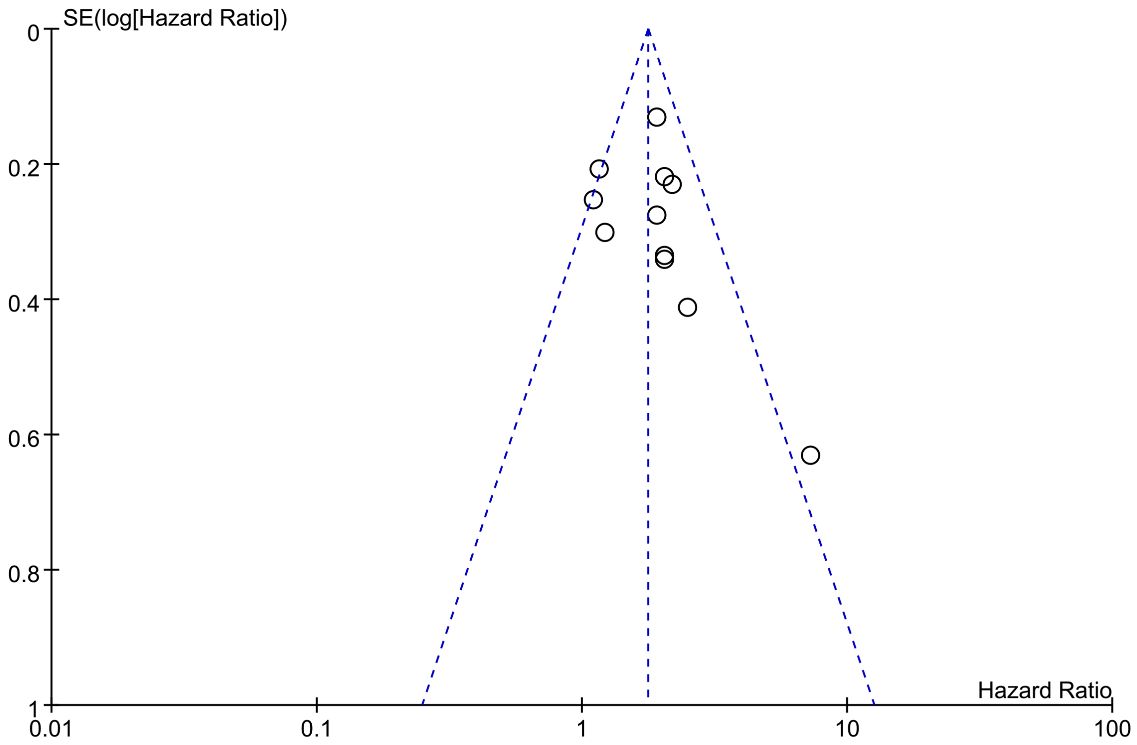

Supplementary Figure S2: Funnel plot of overall survival.

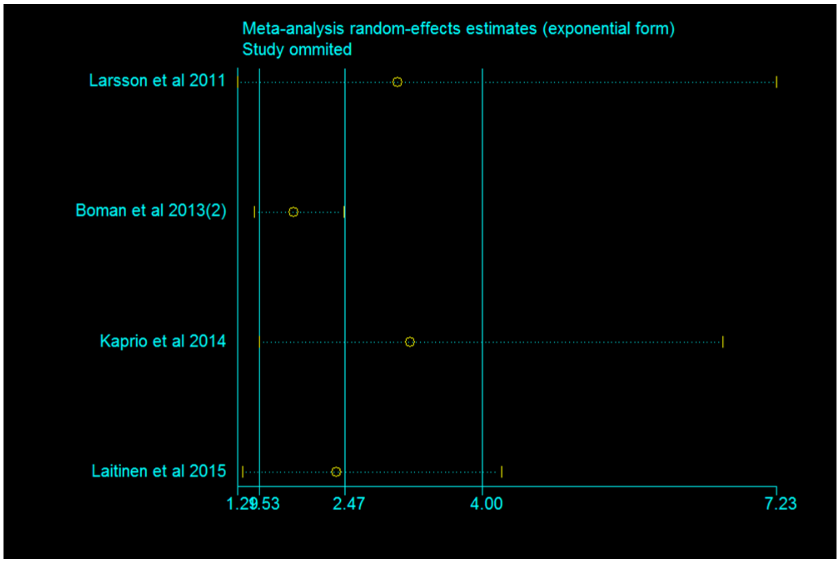

Supplementary Figure S3: Influence analysis of disease-specific survival.

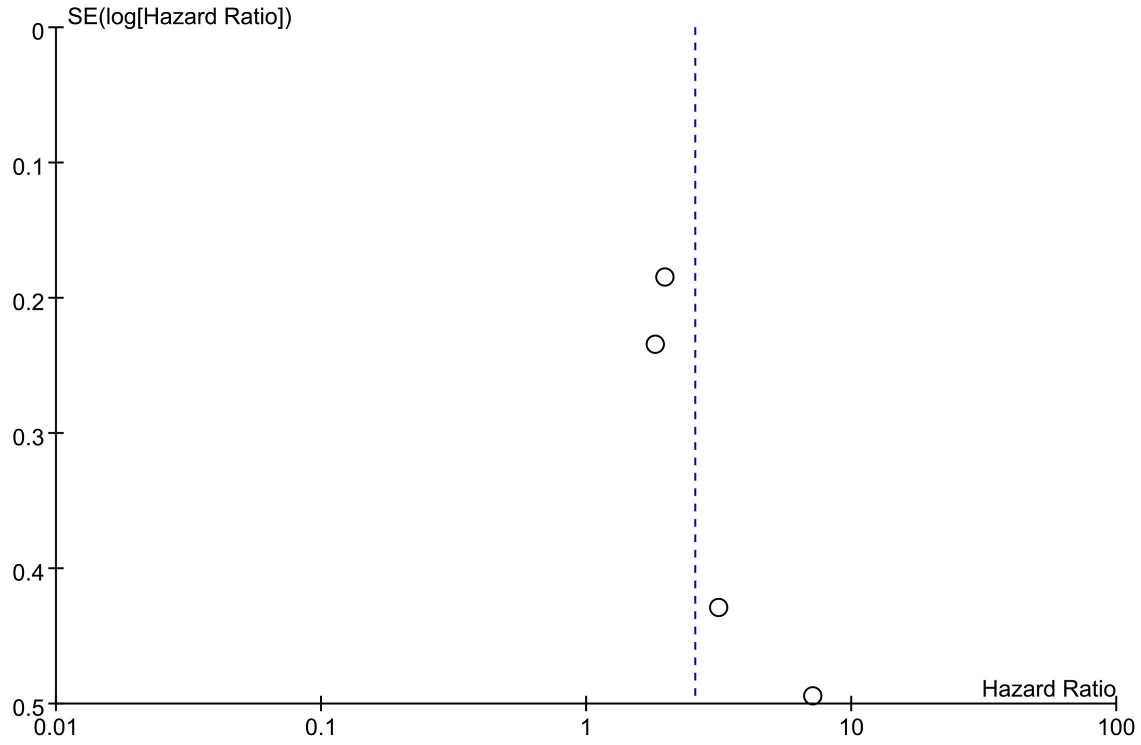

Supplementary Figure S4: Funnel plot of disease-specific survival.

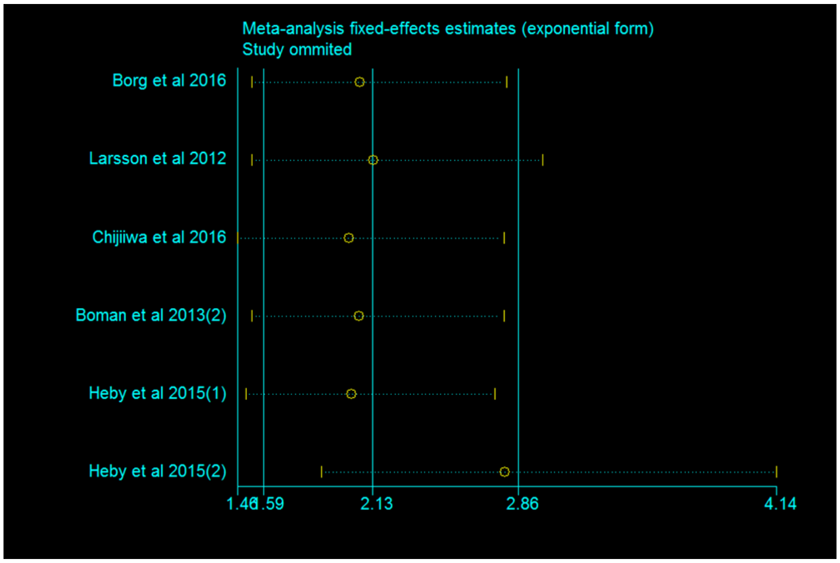

Supplementary Figure S5: Influence analysis of disease free survival.

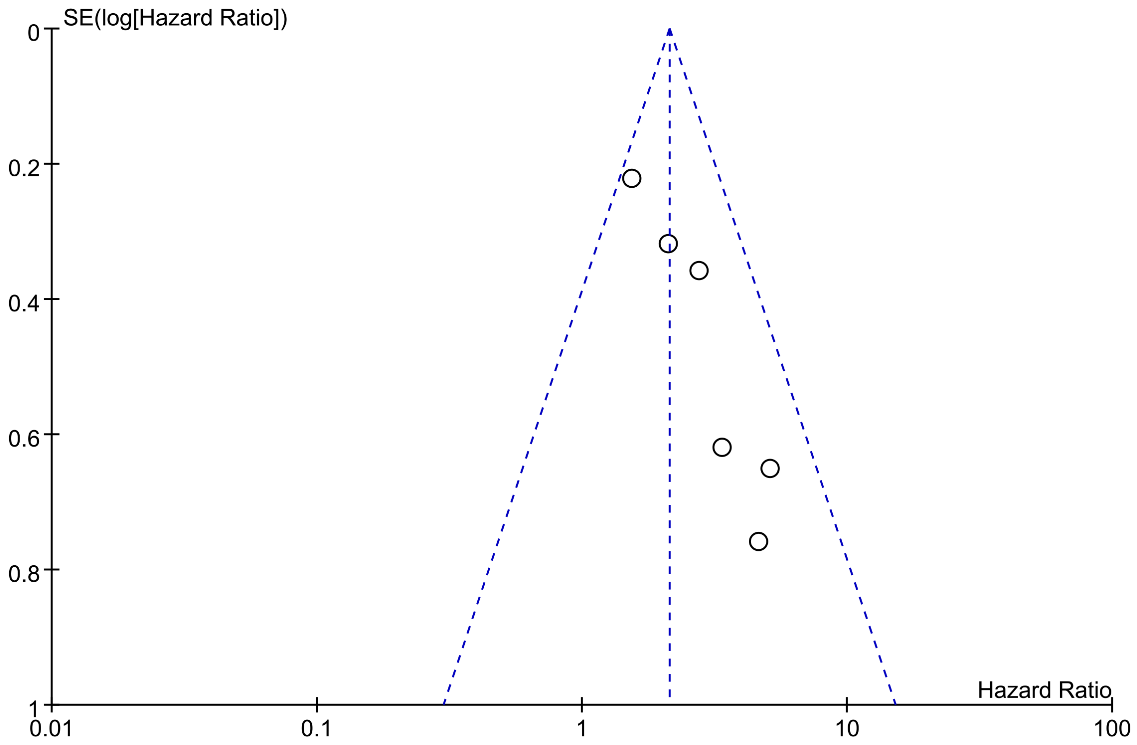

Supplementary Figure S6: Funnel plot of disease free survival.

Supplementary Table S1: PRISMA 2009 Checklist

See Supplementary File 1
